# Impact of Solar Light and Electricity on the Quality and Timeliness of Maternity Care: A Stepped-Wedge Cluster-Randomized Trial in Uganda

**DOI:** 10.9745/GHSP-D-21-00205

**Published:** 2021-12-31

**Authors:** Slawa Rokicki, Brian Mwesigwa, Peter Waiswa, Jessica Cohen

**Affiliations:** a Rutgers School of Public Health, Piscataway, NJ, USA.; b University College Dublin, Dublin, Ireland.; c Innovations for Poverty Action, Kampala, Uganda.; d Maternal, Newborn and Child Health Centre of Excellence, Makerere University School of Public Health, Kampala, Uganda.; e Global Public Health, Karolinska Institutet, Stockholm, Sweden.; f Busoga Health Forum, Jinja, Uganda.; g Harvard T.H. Chan School of Public Health, Boston, MA, USA.

## Abstract

**Background:**

We evaluated the impact of solar light installation in Ugandan maternity facilities on implementation processes, reliability of light, and quality of intrapartum care.

**Methods:**

We conducted a stepped-wedge cluster-randomized trial of the We Care Solar Suitcase, a complete solar electric system providing lighting and power for charging phones and small medical devices, in 30 rural Ugandan maternity facilities with unreliable lighting. Facilities were randomly assigned to receive the intervention in the first or second sequence in a 1:1 ratio. We collected data from June 2018 to April 2019. The intervention was installed in September 2018 (first sequence) and in December 2018 (second sequence). The primary effectiveness outcomes were a 20-item and a 36-item index of quality of intrapartum care, a 6-item index of delays in care provision, and the percentage of deliveries with bright light, satisfactory light, and adequate light.

**Results:**

We observed 1,118 births across 30 facilities. The intervention was successfully installed in 100% of facilities. After installation, the intervention was used in 83% of nighttime deliveries. Before the intervention, providers on average performed 42% of essential care actions and accumulated 76 minutes of delays during nighttime deliveries. After installation, quality increased by 4 percentage points (95% confidence interval [CI]=1,8) and delays in care decreased by 10 minutes (95% CI=−16,−3), with the largest impacts on infection control, prevention of postpartum hemorrhage, and newborn care practices. One year after the end of the trial, 90% of facilities had LED lights in operation and 60% of facilities had all components in operation.

**Conclusions:**

Reliable light is an important driver of timely and adequate health care. Policy makers should invest in renewable energy systems for health facilities; however, even when reliable lighting is present, quality of care may remain low without a broader approach to quality improvements.

## Abstract (precis)

Lack of access to reliable energy is a major neglected health system challenge to maternal and child health. We found that installing a solar energy system intervention in rural Ugandan maternity facilities led to modest increases in the quality of maternity care and reductions in delays in care.

## Teaser

**Key Findings:**

- Universal access to modern energy and safe childbirth are global health priorities that are inextricably linked, yet little research has rigorously examined the adoption and effectiveness of solar energy systems in resource-constrained settings.
- In sub-Saharan Africa, health facilities lack access to reliable light and electricity. We evaluated the implementation and effectiveness of the We Care Solar Suitcase, a complete solar electric system that provides medical lighting and electrical power for charging small medical devices and mobile phones, on the quality of maternal and infant care.
- The intervention was adopted well by health providers, increased facility brightness, and led to modest increases in the quality of maternal care based on clinical observations of deliveries.
- Reliable light is an important driver of timely and adequate health care and may improve providers' ability and timeliness in performing actions needed to reduce the risk of postpartum hemorrhage.

**Key Implications:**

Ministries of health, development partners, and other health care reform stakeholders should:

- Invest in modern and renewable energy systems for health care facilities.
- Ensure access to reliable energy is included in larger efforts to improve quality of care via systemic changes to health systems.

## BACKGROUND

Reducing maternal and neonatal mortality are global public health priorities, yet progress on these goals remains intractably slow. The burden of maternal mortality in low-income countries is staggering—if countries could meet the Sustainable Development Goal of 70 maternal deaths per 100,000 live births by 2030, the lives of an estimated 1.6 million mothers would be saved.^1^ With dramatic increases in facility-based deliveries over the past 30 years, the major barrier to meeting this target now lies not in increasing access to health facilities, but in improving the quality of care delivered in these facilities.^2^^,^^3^ A recent analysis found that over half of maternal and neonatal deaths in low-income countries result from poor-quality care rather than from nonutilization of care.^3^ High-quality care requires the provision of effective, timely, and safe health services, delivered by a well-trained and motivated workforce, in a facility equipped with essential infrastructure and supplies, functioning health information systems, and good leadership and governance.^4^

One of the major neglected health system challenges to maternal and child health is the lack of access to reliable energy. Reliable light and electricity are critical for nearly all aspects of safe childbirth, including equipment sterilization, infection control, powering essential medical devices, and nighttime examinations and procedures.^5^^,^^6^ Lack of reliable light may hinder providers' ability to manage complications and cause them to delay necessary care actions, putting both mother and infant at risk.^7^ Frequent blackouts may also create stressful working conditions for health care workers, generate patient mistrust, and promote inequities in care.^8^^,^^9^ Yet a study across 78 low- and middle-income countries (LMICs) found that 59% of health facilities lack access to reliable electricity.^10^ In sub-Saharan Africa, one-quarter of health facilities have no connection to the electrical grid; among connected facilities, frequent and prolonged interruptions to power are common.^11^ This crisis has been compounded by the health and economic consequences of the coronavirus disease (COVID-19) pandemic, a time when proper infection control in health facilities is imperative to mitigate the spread of the virus.

The significance of access to affordable, reliable, and modern energy in strengthening health systems has been recognized by the United Nations' Sustainable Energy for All (SEforAll) and United States Agency for International Development (USAID) Powering Health Care initiatives.^12^ While improved sources of reliable lighting are needed in many health systems in LMICs, there are significant barriers to expanding grid power to unconnected health facilities, including the high cost per mile to extend and maintain a grid to remote areas and the strain such extensions would place on already weak and unreliable infrastructure. Moreover, recent experimental evidence on the demand for rural residential grid connections has found low willingness to pay for electrification among residents; however, aligning programs for new grid connections with opportunities for productive use (e.g., developing small businesses) may facilitate a higher willingness to pay.^13^^,^^14^

While improved sources of reliable lighting are needed in many health systems in LMICs, there are significant barriers to expanding grid power to unconnected health facilities.

Renewable energy sources such as solar power may provide a clean, efficient, and cost-effective opportunity to increasing access to reliable light and electricity for health facilities in resource-constrained settings.^5^ However, there are several challenges to the implementation of solar energy systems in rural health facilities. Health care workers may be reluctant to adopt new technology if they do not see an immediate benefit, or they may use it inconsistently or incorrectly. This outcome is a common finding in the implementation of mobile health and electronic health registration systems in LMICs.^15^^–^^18^ Poorly designed technology can quickly fall into disrepair, while inadequate maintenance, such as lack of replacement of batteries, can result in disuse. Finally, there is difficulty in sustainability and scale-up.^19^ Evidence is needed on whether adopting and integrating solar energy into rural health facilities (1) is feasible, (2) improves reliability and brightness of light, and (3) affects quality of care. Most prior quantitative studies examining the relationship between reliable light and health care quality are observational and are subject to unobserved confounding related to patient, provider, and facility factors. As far as we are aware, the only randomized trials evaluating the impact of providing electricity or light on health outcomes in LMICs have included electricity upgrades as part of a broader package of infrastructure, training, supervision, and mentoring, complicating any inference about the specific role that electrification and bright light play in quality of care and patient outcomes.^20^^–^^22^ Further, detailed data on light and quality of care—for example, through actual observations of light and health care worker actions—are rarely available.

We conducted a stepped-wedge cluster-randomized controlled trial in Uganda to evaluate the impact of implementing the We Care Solar Suitcase, a complete solar electric system that provides medical lighting and electrical power for charging small medical and communication devices. We first examine the implementation of the intervention in health facilities. We then assess the extent to which using the Solar Suitcase improves light brightness using light sensors and direct observations of light sources. Finally, we use clinical observations of care to evaluate the benefit of the solar system across a range of clinical processes, including the provision of adequate care and the timeliness of care received.

We assess the extent to which using the We Care Solar Suitcase improves light brightness using light sensors and direct observations of light sources.

**Figure:**
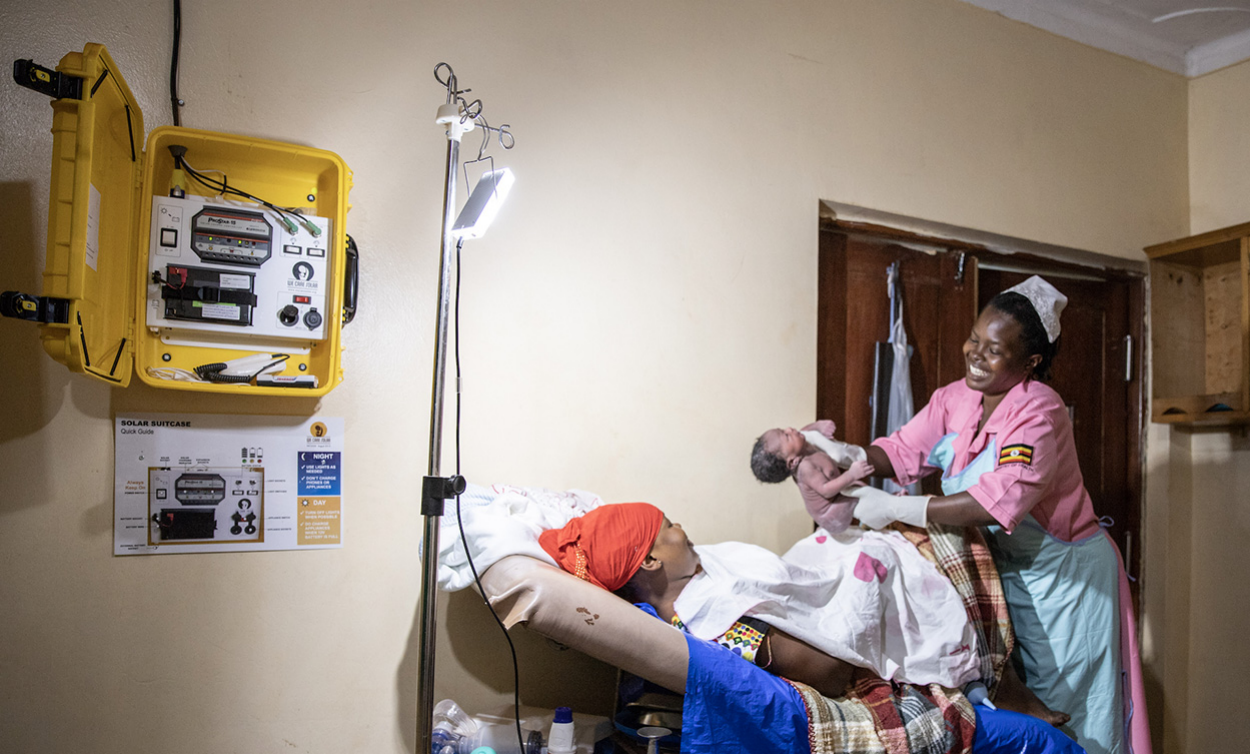
Asiat Musenze, a Ugandan midwife, attends to a mother and her newborn with the light of the Solar Suitcase. © 2018 Zahara Abdul

## METHODS

### Study Setting

The study took place in maternity facilities in Uganda. From 2011–2016, the maternal mortality ratio for Uganda was 336 deaths per 100,000 live births, while the perinatal mortality rate was 38 deaths per 1,000 pregnancies.^23^^,^^24^ In 2016, 73% of deliveries in Uganda occurred in a health facility.^23^

Uganda's health sector hierarchy includes national and regional hospitals, followed by a district health system composed of health centers of levels II through IV. These health centers generally staff low- and mid-level providers who provide care for uncomplicated deliveries.

### Study Design

The study design was a stepped-wedge cluster-randomized controlled trial as a staggered rollout of the intervention was necessary owing to limited resources. Facilities were randomized into 1 of 2 sequences, with the first sequence receiving the intervention between the first and second periods of data collection, and the second sequence receiving the intervention between the second and third periods of data collection (Supplement Figure 1). Details of the study methods have been published in the study protocol.^25^

### Participants

Clusters were primary health facilities in the Central, Eastern, and Western regions of Uganda. We conducted a census of all primary health facilities offering delivery services in these regions. We then excluded facilities that did not meet the inclusion criteria. Details of inclusion and exclusion criteria are specified in Supplement Table 1. In brief, criteria for facilities were (1) level II, III, or IV; (2) open 24 hours a day; (3) the presence of unreliable overhead light; (4) no automatic referral of women elsewhere during blackouts; and (5) willingness of the medical officer in-charge to participate. All health care workers that worked in the maternity ward at these facilities were eligible for interview and observation. Pregnant women aged 16 and older who were admitted to the facilities for labor and delivery and provided written informed consent were eligible to participate.

### Randomization

Randomization of facilities was conducted using Stata's randomize command, with stratification by geographic cluster and a measure of baseline light availability. The randomization that achieved the best balance based on baseline quality of care and facility volume was chosen (Supplement Table 2 has details on stratification and balancing variables). The allocation sequence was generated by the study investigators. Due to the nature of the intervention, neither participants nor researchers were blinded to allocation.

### Ethical Approval

The study was approved by Institutional Review Boards at the Harvard T. H. Chan School of Public Health, the Mildmay Uganda Research Ethics Committee, and the Uganda National Council for Science and Technology. Written informed consent was obtained from the facility in-charge or head, all maternal care providers, and all women older than 16 years of age that presented for normal deliveries at facilities.

### Intervention

The intervention was the We Care Solar Suitcase, a complete solar electric system that provides medical lighting and electrical power for charging phones and small medical devices. The system contains a photovoltaic solar panel installed on the roof of a health facility; a 12-V lithium ferrous phosphate battery; high-efficiency, moveable light-emitting diode (LED) lights for maternity rooms; and 2 rechargeable LED headlamps. In addition, it contains a fetal Doppler with rechargeable batteries, 2 12-V DC accessory sockets, 2 USB ports for charging cell phones, and an AA/AAA battery charger. Installations were conducted by a local solar contracting firm based in Uganda. One Solar Suitcase was installed in each facility, with 2–4 overhead LED lights for each delivery room, depending on its size. The product specifications for the Solar Suitcase are open source and technical details are provided in the Supplement Figure 2 (version 2.0) and Figure 3 (version 3.0). Version 2.0 was used in this study. The cost of building a Solar Suitcase, including parts and manufacturing, is US$2,220 (2018 value). If parts wear out or break down, We Care Solar works with implementation partners, government partners, and on-the-ground staff to identify appropriate in-country recycling and disposal facilities.

To ensure consistent and appropriate use of the installed Solar Suitcase, installers conducted trainings with health care workers on how to use and maintain it and all of its accessories on the day of installation and in subsequent check-ins, as needed. The cost of installation and training is US$250 per Solar Suitcase.

Data collection began in June 2018 and was completed in April 2019. The intervention was installed in September 2018 (first sequence) and in December 2018 (second sequence).

### Data Collection

Quality of care was measured with direct clinical observations of deliveries using an extensive clinical observation tool adapted from the Maternal and Child Health Integrated Program (MCHIP) Quality of Care Surveys.^26^ Using this observation tool, enumerators indicated whether items that are essential for high-quality care were provided during labor, delivery, and the early postpartum period. Enumerators observed and recorded the care delivered by providers during labor and delivery over 4 stages: arrival and first examination, first stage of labor, second and third stages of labor, and the first hour postpartum. For each item in the observation tool, enumerators recorded whether the item was completed by a health care worker and, if it was completed, recorded a timestamp as to when the item was completed. In addition, enumerators indicated the sources and brightness of light at each of the 4 stages of the observation. The brightness was recorded as “very bright,” “somewhat bright,” “dim,” and “pitch black.” Definitions for the level of brightness for these categories are provided in Supplement Table 3; enumerators were trained to interpret these categories uniformly according to these definitions. Finally, after an observation was complete, enumerators reviewed the patient's chart to retrieve information on patient age, parity, and gestational age.

To reduce disruption and influence, enumerators were trained to avoid interaction with providers and patients. Enumerators were provided with digital watches for recording timestamps. To maximize interrater reliability, enumerators were extensively trained on the definition of each item in the observation checklist under the leadership of the study obstetrician. Details on enumerator protocol and interrater reliability are provided in Supplement Table 2.

Data collection was conducted on paper questionnaires, then inputted electronically using double data entry. A Stata user-written code file was used to identify discrepancies between entries, which were resolved by the project manager by referring to the original paper questionnaire and/or by contacting the enumerator. Data management procedures were in place to ensure data quality, including daily checks by the project manager on incoming data to identify data outliers, logical inconsistencies, and missing data.

At the end of each data collection period, enumerators conducted provider interviews to record information on provider demographics, work experience, training, and attitudes. Enumerators also conducted facility assessments with the medical officer in charge to record information on facility staff, monthly patient volume, and electricity interruptions.

Finally, light sensors were installed (HOBO 4-Channel Analog data logger) in the delivery rooms of each facility that recorded the light (measured in lux) at each minute for the duration of the study.

Data collection tools were piloted at 5 facilities before study rollout to ensure usability, clarity, and inter-enumerator reliability. Light sensors were also piloted to ensure correct implementation procedures and data usability. Data from these pilot facilities were not included in the final sample.

### Implementation Outcomes

We applied the RE-AIM framework to guide evaluation of implementation outcomes.^27^ We assessed the reach of the intervention by examining the percentage of eligible facilities that participated in the study, as well as the representativeness of study facilities and women delivering in facilities by comparing facility and participant descriptive characteristics with national-level estimates. We examined the implementation of the intervention by determining the percentage of facilities with successful installations and the percentage of health care workers successfully trained. We examined adoption via enumerator-reported main source of light used during the delivery observation before and after implementation of the Solar Suitcase. Sources of light included the Solar Suitcase, electrical grid, other overhead solar light, generator, kerosene lamp, flashlight, and daylight. We also examined whether health care workers self-reported that they felt comfortable using the components of the Solar Suitcase (binary measure) and how often they reported using the various components (lights, headlamp, and fetal Doppler) on a 5-point Likert scale. We examined maintenance by the percentage of facilities with Solar Suitcase components still in operation at 3 post-trial follow-up visits, ranging from 5 months to 1 year after the completion of the trial.

### Effectiveness Outcomes

The primary effectiveness outcomes pre-registered on ClinicalTrials.gov include measures of light, quality of care, and health care worker satisfaction. This article reports on the light and quality of care outcomes. Health care worker outcomes will be reported in a separate paper.

We examined changes in reliability and brightness of light in several ways. We constructed a binary variable for “bright light,” which was equal to 1 if the room was “very bright” or “somewhat bright” throughout the entire labor and delivery observation and 0 if the room was “pitch black” or “dim” at any point during observation. The rationale for this breakpoint was that providers were not able to adequately provide care in conditions that were dim or pitch black, according to the definitions for these categories. We also constructed a binary variable for a “satisfactory light source,” which was equal to 1 if, throughout the entire observation, one of the following overhead sources was used: Solar Suitcase, electrical grid, other overhead solar light, generator, or daylight. This variable was equal to 0 if any of the following light sources were used during the observation: kerosene lamp, candle, flashlight, solar lamp, or no light at all. The satisfactory sources of light were defined as overhead lights that light the entire room, as opposed to ground-level lights that must be moved to see different areas of the room and only light a small area. The rationale was that moving and holding a light source is dangerous as it occupies and contaminates a health care worker's hands; moreover, unsatisfactory sources such as kerosene lamps and candles are fire hazards and contribute to indoor air pollution. We also combined the source and brightness of the light into a variable indicating “adequate light,” which was defined as a binary variable equal to 1 if the light was from a satisfactory source and was “bright” (“very bright” or “somewhat bright”) for the duration of observation.

Finally, light sensors were used to measure the number of minutes of light and the level of brightness during the day and night (details on light measures are provided in Supplement Table 3).

Quality of care was measured via enumerator observation by extracting 2 indices of quality and 1 index of delays in care from the extended MCHIP observation tool. First, we used a 20-item quality of care index developed for and validated in low- and middle-income settings.^28^ The index is composed of 20 indicators representing essential components of the process quality of intrapartum and immediate postpartum care in facility deliveries, between the initial patient assessment and first hour postpartum (Supplement Table 4 has individual items). Second, we extended this index to include an additional 16 items to create a 36-item index, with additional items adapted from the MCHIP tool.^26^ The longer index captures additional items that may be particularly affected by the Solar Suitcase, such as checking the fetal heart rate and disposal of waste. Both indices were constructed as the percentage of total items performed per delivery observation and thus range from 0% to 100%. We also calculated section indices across areas of (1) history taking/communication, (2) patient assessment, (3) infection control, (4) prevention of postpartum hemorrhage, and (5) newborn care.

Quality of care was measured via enumerator observation, using 2 indices of quality and 1 index of delays in care from the extended MCHIP observation tool.

Lastly, we constructed a “delays in care” index, capturing items that occur throughout the delivery process, including time between facility arrival and first contact with health care worker, time between facility arrival and first vaginal examination, time between delivery and provision of uterotonic, time between delivery and assessment of perineal and vaginal lacerations, time between delivery and drying baby with towel, and time between delivery and initiation of breastfeeding. Measurements and definitions of outcomes are provided in more detail in Supplement Table 3.

### Statistical Analysis

Results from baseline (period 1) were used to provide initial estimates on power and sample size. Sample size calculations were conducted using the stepped-wedge function in Stata v15. We estimated the detectable effect sizes assuming 22 births per facility (average cluster size) for 2 steps, 13 clusters randomized at each step, 80% power, and α=0.05. We assumed, conservatively, that 61% of deliveries before the intervention would be conducted without adequate light, with an intracluster correlation (ICC) of 0.20. Our minimum detectable effect size for adequate light was 13 percentage points. For quality, we assumed an average pre-intervention score of 44%, with an ICC of 0.4. Our minimum detectable effect size based on these conservative estimates was 11 percentage points. In practice, our observed cluster size was 38 births per facility, which led to a larger detectable effect size than estimated in power calculations.

We first conducted an analysis of the causal effect of the intervention on outcomes using linear regression models with facility fixed effects. The parameter of interest was the coefficient on an indicator for whether the observation occurred in a facility that had been randomized to receive the Solar Suitcase installed at the time of observation or not. To account for the varying amount of time spent observing at each facility (due to differences in patient volume and availability of light), models were also adjusted for the duration of time spent in facilities (measured by the number of enumerator shifts worked at each facility in each period). Standard errors were clustered at the facility level.

We analyzed all observations as well as the subset of observations in which at least some part of the observation occurred during the nighttime hours of 6:00 pm to 7:00 am. Our rationale was that, while clinical care provided during nighttime hours would likely be the most directly affected by the adequacy of light, facility lighting may be dim and could benefit from brighter light even in daytime hours.

In our analysis, we first present the sources of light used to conduct deliveries during nighttime hours (6:00 pm to 7:00 am) before and after the intervention, presenting the light source at the time the newborn was delivered. We next present the impact of the intervention visually, plotting linear predicted values over period and sequence from the regression models described above. Finally, we present regression results.

### Sensitivity Analysis

We conducted several sensitivity analyses, including alternate specifications, adjustment of standard errors using the wild cluster bootstrap method,^29^^,^^30^ imputation of missing data, assessment of enumerator bias or Hawthorne effects,^31^ assessment of whether there was selection of providers or patients into better lit facilities after implementation, and the inclusion of observations that resulted in a multiple birth, stillbirth, or infant death (Supplement Table 5 includes methodological details of sensitivity analyses).

### Patient and Public Involvement

This research was done with guidance from of the Uganda Ministry of Health, Directorate of Clinical Services, which provided authorization to conduct the research in particular districts, as well as input into health facility selection. At a district level, input on facility selection and support for the study were provided by district health officers. Results will be disseminated to the Ministry of Health.

## RESULTS

### Reach

**FIGURE 1:**
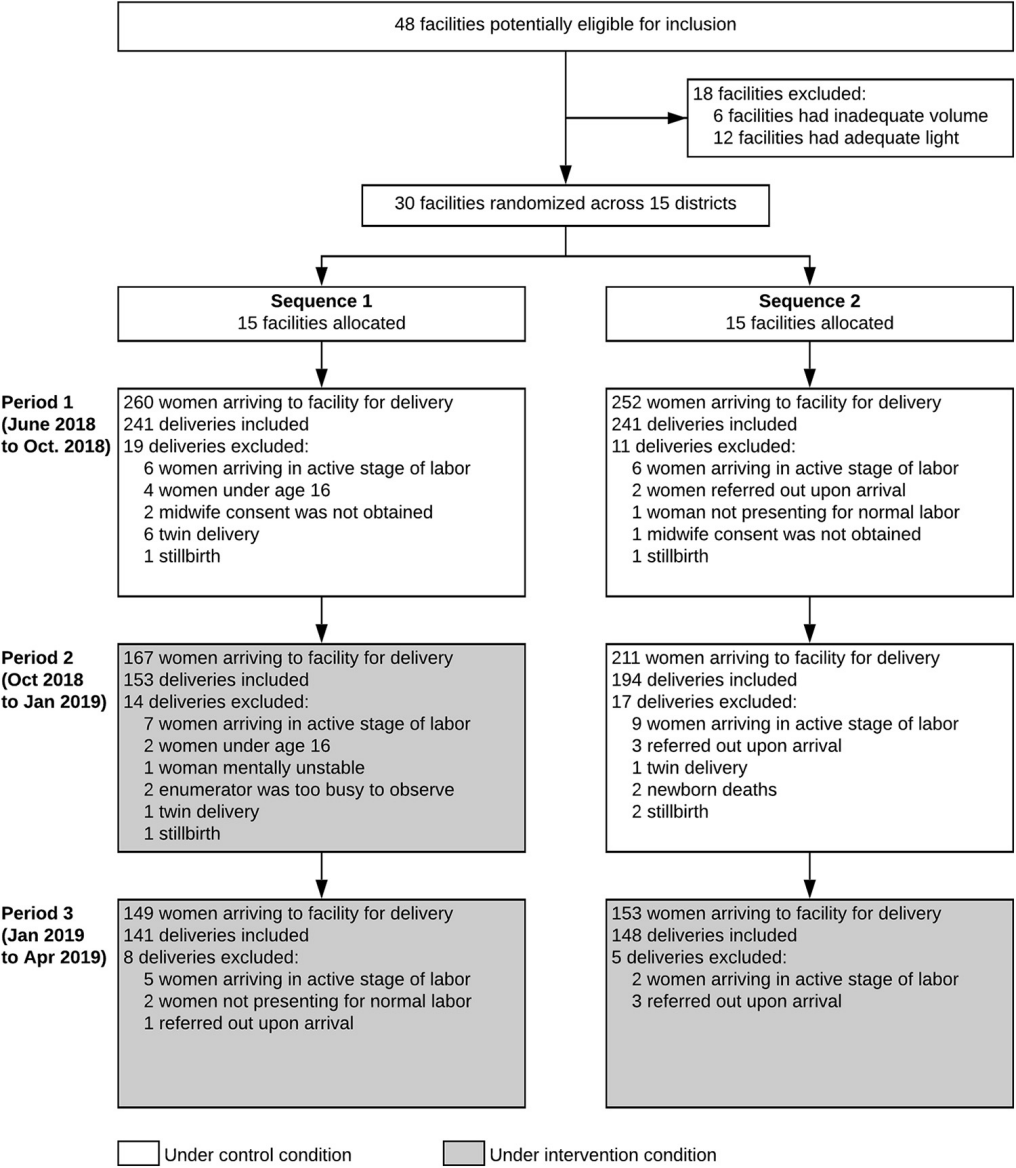
Consort Diagram of Maternal Health Care Facilities in Uganda Included in the Solar Suitcase Intervention Study

All facilities that met the inclusion criteria agreed to participate in the study and all health care workers within these facilities eligible for participation also agreed to participate (Figure 1). Facilities included in the study were similar to primary-level facilities in Uganda. In the most recent nationally representative survey of health facilities in Uganda (2013), 84% of primary facilities offer 24-hour facility-based delivery services and 51% have unreliable electricity.^32^

Between June 2018 and April 2019, 1,183 patients arrived at study facilities to deliver. Of these, 59 were excluded at arrival, with the most common reason being that the patient arrived in the active stage of labor so that written consent was not possible to obtain before delivery. Additionally, we excluded 8 observations that were twin deliveries, 2 that resulted in a newborn death, and 5 that resulted in a stillbirth. The final sample size was 1,118 birth observations.

Table 1 shows pre-intervention characteristics of facilities, providers, and patients, as well as tests for balance in these characteristics across the facilities randomized to receive the intervention first (sequence 1) and the facilities randomized to receive the intervention second (sequence 2). No systematic evidence of imbalance was apparent, with the *P*-value for the *F*-test on joint equality equal to 0.24. The mean number of staff present was 7 in our sample, compared with 8 in the national survey sample of primary-level facilities.^33^ On staff, we observed more midwives/nurses (70%) than clinical officers (15%) and nursing assistants (15%), compared with 44% midwives/nurses, 13% clinical officers, and 44% nursing assistants in the national survey. Facilities in the sample also experienced higher patient volume (34 average deliveries per month) compared with the national sample (14 average deliveries per month). Women delivering were an average of 24.5 years old, with a parity of 2.3, and an average gestational period of 38.1 weeks. In comparison, a national sample of pregnant women in Uganda had an average age of 25.8, with a parity of 1.2.^23^

**TABLE 1.** Baseline Characteristics of Maternity Health Care Facilities in Uganda (N=30)^a^ Included in the Study of the Solar Suitcase Intervention on the Quality of Intrapartum Care

	Overall	Sequence 1	Sequence 2
	N=30	n=15	n=15
No. of observations per facility, mean (SD)	38 (16)	36 (19)	39 (14)
No. of days spent observing, mean (SD)	30 (12)	30 (12)	30 (12)
No. of MCH staff employed, mean (SD)	7 (4)	6 (3)	8 (5)
Monthly patient volume, mean (SD)	34 (17)	30 (18)	37 (16)
Primary source of electricity, n (%)			
None/lanterns	12 (40)	6 (40)	6 (40)
Grid	11 (37)	5 (33)	6 (40)
Solar	7 (23)	4 (27)	3 (20)
Facility government owned, n (%)	28 (93)	13 (87)	15 (100)
Facility level, n (%)			
Health center II	5 (17)	3 (20)	2 (13)
Health center III	22 (73)	11 (73)	11 (73)
Health center IV	3 (10)	1 (7)	2 (13)
Provider age, mean (SD)	34 (5)	33 (5)	35 (6)
Provider years of experience, mean (SD)	8 (6)	7 (5)	9 (7)
Proportion of providers with secondary education, mean (SD)	0.02 (0.07)	0.04 (0.10)	0.00 (0.00)
Proportion of providers with certificate, mean (SD)	0.60 (0.32)	0.62 (0.35)	0.58 (0.31)
Proportion of providers with diploma, mean (SD)	0.38 (0.32)	0.34 (0.34)	0.42 (0.31)
Proportion of providers in officer position, mean (SD)	0.15 (0.22)	0.15 (0.22)	0.16 (0.22)
Proportion of providers in midwife/nurse position, mean (SD)	0.70 (0.26)	0.61 (0.28)	0.79 (0.23)
Proportion of providers in nursing assistant position, mean (SD)	0.15 (0.22)	0.24 (0.26)	0.06 (0.12)
Quality score, mean (SD)	44.2 (8.4)	43.5 (7.9)	45.0 (9.0)
Delay index score (minutes), mean (SD)	73.5 (16.9)	76.4 (19.4)	70.7 (14.1)
Proportion of adequate light throughout observation, mean (SD)	0.44 (0.21)	0.47 (0.22)	0.41 (0.20)
Mother's age (years), mean (SD)	24.5 (2.5)	25.0 (2.8)	24.1 (2.1)
Mother's parity, mean (SD)	2.3 (0.6)	2.3 (0.5)	2.3 (0.7)
Gestational age (weeks), mean (SD)	38.1 (0.9)	38.0 (0.9)	38.1 (0.9)
Overall *F*-test	0.24		

Abbreviations: MCH, maternal and child health; SD, standard deviation.

a The overall F-test is a joint test of orthogonality of all variables. Quality score is the percentage of items performed of the 20-item index. Delay index score is the sum of 6 items in the delays index (Supplement Table 3).

### Implementation and Adoption

**FIGURE 2:**
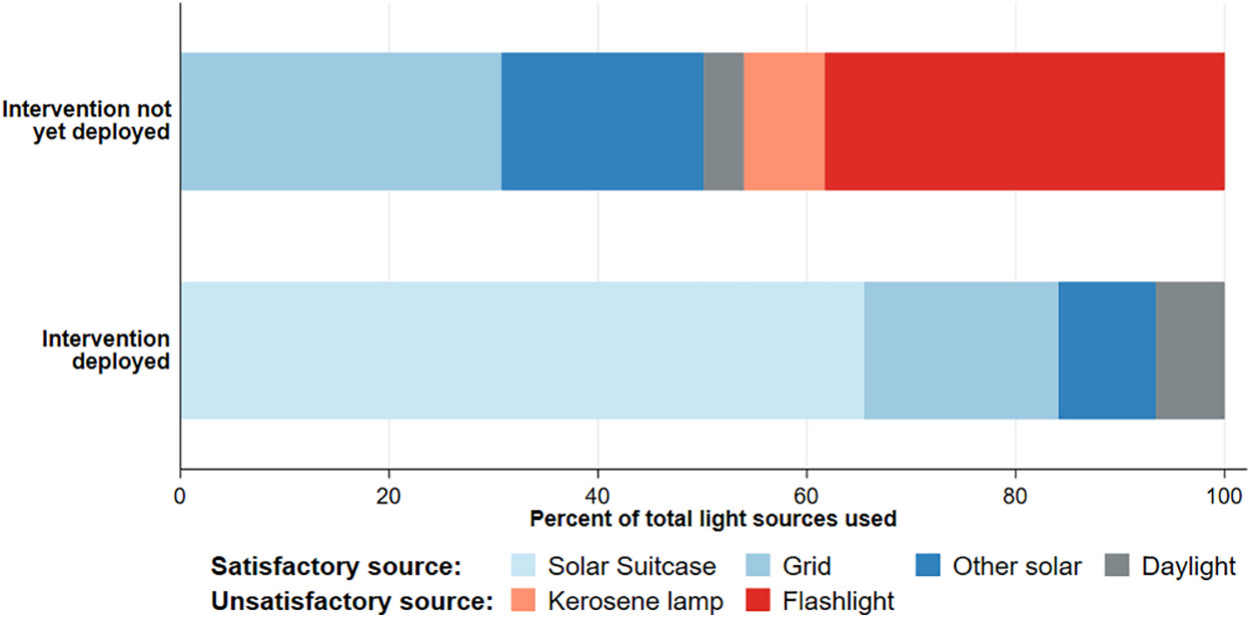
Source of Light During the Delivery of Infant, in Periods When Facilities Had a Solar Suitcase Compared With Periods When Facilities Did Not Have a Solar Suitcase, Among Observations in Which Birth Occurred During Nighttime Hours^a^ ^a^ Sources of light during the actual delivery are shown, as the percentage of the number of times each source is used out of the total number of light sources used across all nighttime deliveries. Nighttime hours refer to between 6:00 pm and 7:00 am. Figure shows only sources that made up more than 3% of observations (dropped sources include solar lantern, candle, and darkness).

Over the course of the trial, the Solar Suitcase intervention was successfully installed in 100% of facilities, with 76% of health care workers trained on its use by the contractor and another 13% trained by another health care worker in the facility. Figure 2 shows adoption of the Solar Suitcase into the health facilities by examining sources of light used to conduct deliveries during nighttime hours (6:00 pm to 7:00 am) before and after the intervention, as measured by direct observation. Across all deliveries that occurred during nighttime hours (n=571), there were 690 light sources used; 20% of deliveries used more than 1 light source. Before the intervention, 44% of these light sources used were either a kerosene lamp or flashlight, compared with 0% of light sources used after the intervention was deployed. After it was deployed, the Solar Suitcase was used in 83% of all nighttime deliveries and made up 65% of all light sources used.

The Solar Suitcase was successfully installed in 100% of facilities, with 89% of facility health care workers receiving training on its use.

Health care workers' self-reported use of the Solar Suitcase was consistent with the results from direct observation shown in Figure 2. Ninety percent of health care workers agreed that they felt comfortable using the Solar Suitcase and 88% of health care workers used the Solar Suitcase LED lights for most or every nighttime delivery, although less than 10% of health care workers used the Solar Suitcase lights during the day. Use of the headlamp and fetal Doppler components were less consistent: 56% of health care workers never or rarely used the headlamp, while 29% never or rarely used the fetal Doppler (Supplement Table 6).

### Effectiveness

**FIGURE 3:**
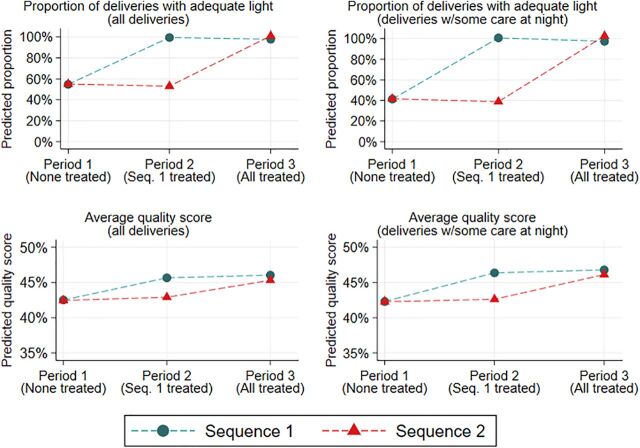
Impact of Solar Suitcase Intervention on Adequacy of Light and the 20-Item Quality of Care Index, for all Observed Deliveries (n=1,118) and for Observed Deliveries With Some Nighttime Hours (n=743) in Maternal Health Care Facilities in Uganda^a^ ^a^ “Adequate light” indicates that all observed parts of observation were perceived to be under a bright light and from a satisfactory light source. Quality score is the percentage of items performed of the 20-item Tripathi index. Supplement Table 3 has detailed definitions of variables. Results from predicted margins of linear regression with facility fixed effects. Nighttime hours refers to between 6:00 pm and 7:00 am.

Figure 3 presents the graphic representation of the impact of the intervention on measures of adequate light and quality of care. The figure demonstrates that sequence balance was achieved in period 1 for both adequate light and quality outcomes. While the proportion of all deliveries with adequate light was the same across sequences in period 1 at 56%, this proportion increased to 100% in period 2 for facilities in sequence 1, while it remained stagnant for facilities in sequence 2. In period 3, both sequences indicated 100% of deliveries were conducted with adequate light. The results on quality have a similar trapezoidal shape in the figure, providing strong evidence that the changes in outcomes can be attributed to the intervention.

We formalize this descriptive analysis with the results of the regression models (Table 2). Before the intervention, 56.1% of all observed deliveries (42.4% of deliveries with some nighttime hours) were conducted with bright light and 66.3% of all deliveries (55.7% of deliveries with some nighttime hours) were conducted with a satisfactory light source (Table 2). Similarly, 54.4% of deliveries were conducted with adequate light (41.0% of deliveries with some nighttime hours). The intervention had a large and significant impact on all light measures, increasing the proportion of all deliveries conducted with bright light by 43.7 percentage points (95% CI=35.9, 51.5), the proportion with a satisfactory light source by 33.3 percentage points (95% CI=27.2, 39.4), and the proportion with adequate light by 45 percentage points (95% CI=37.2, 52.8). Estimates were larger for the subset of deliveries with some nighttime hours. These results indicate that after the intervention, 100% of all deliveries and nighttime deliveries were conducted with a bright, satisfactory light source and adequate light. The calculated ICCs were generally lower than assumed.

**TABLE 2.** Linear Estimates of the Impact of the Solar Suitcase Intervention on Light and Quality of Intrapartum Care^a^ in Maternal Health Care Facilities in Uganda

Outcome	N	ICC	Average before intervention, %	Average after intervention, %	Difference, % (95% CI)
All observed deliveries					
I. Light brightness and source					
Deliveries with bright light	1,118	0.07	56.1	100	43.7 (35.9, 51.5)
Deliveries with satisfactory light source	1,118	0.07	66.3	100	33.3 (27.2, 39.4)
Deliveries with adequate light	1,118	0.07	54.4	99.4	45 (37.2, 52.8)
II. Quality of care					
20-item quality index	1,118	0.21	42.6	45.7	3.1 (−0.04, 6.24)
36-item quality index	1,118	0.21	53.9	58.1	4.2 (1.47, 6.98)
6-item delays index, min	805	0.12	74.3	63.1	−11.24 (−16.47, −6.01)
Observed deliveries with some nighttime hours					
I. Light brightness and source					
Deliveries with bright light	743	0.14	42.4	100	57.5 (46.0, 69.1)
Deliveries with satisfactory light source	743	0.17	55.7	100	44.7 (35.4, 53.9)
Deliveries with adequate light	743	0.14	41.0	100	59 (47.6, 70.4)
II. Quality of care					
20-item quality index	743	0.22	42.3	46.5	4.1 (0.57, 7.68)
36-item quality index	743	0.22	53.9	58.5	4.7 (1.58, 7.78)
6-item delays index, minutes	575	0.13	76.0	66.3	−9.67 (−16.06, −3.29)

Abbreviations: 95% CI, 95% confidence interval; ICC, intracluster correlation.

a Results show point estimates and 95% confidence intervals. Standard errors are clustered at the facility level. The constant refers to the mean outcome in the preperiod. Nighttime hours refer to hours between 6:00 pm to 7:00 pm. Bright light indicates enumerator reported “perfectly bright” or “bright” (as opposed to “dim” or “dark”) across all 4 sections of the observation. Satisfactory light source indicates that the light source during all 4 sections was either daylight, the grid, solar, or a generator. Adequate light indicates that all 4 sections of the observations used a satisfactory light source and were reported to be bright. Quality of care indices are defined in Supplement Table 3. Delays index is missing for observations in which any one of the 6 delays items is missing.

After the intervention, 100% of all deliveries and nighttime deliveries were conducted with a bright, satisfactory light source and adequate light.

Results of the analysis of sensor data were consistent with enumerator-reported outcomes on light (Table 3). The intervention significantly increased the number of daily minutes of light by 141 minutes (95% CI=7.6, 274.3), from 856 minutes to 997 minutes. While the level of brightness was not significantly affected by the intervention during the daytime hours, the level increased by 10.1 percentage points (0.71, 19.6) during nighttime hours, from 14.5 to 24.7.

**TABLE 3.** Linear Estimates of the Impact of the Solar Suitcase Intervention in Maternal Health Care Facilities in Uganda on Objective Measures of Light Using Sensor Data^a^

Outcome	Average Before Intervention	Average After Intervention	Difference (95% CI)
Minutes of light per 24 hours	856	997	141.0 (7.6, 274.3)
Level of brightness during daytime (7:00 am to 6:00 pm)	47.9	54.2	6.3 (−2.2, 14.8)
Level of brightness during nighttime (6:00 pm to 7:00 am)	14.5	24.7	10.1 (0.71, 19.6)

Abbreviation: 95% CI, 95% confidence interval.

a Results show point estimates and 95% confidence intervals. Standard errors are clustered at the facility level. Regression controls for month of year and facility fixed effects. One facility (in Sequence 2) had a broken sensor and was not included. The number of minutes of light per day is calculated as the number of minutes over a threshold of 20% of the maximum seen in that facility. Results robust to using any threshold between 1% and 35% (results not shown). Level of brightness is on a 0–100 scale, as the percentage of the maximum light the sensor in each facility could read.

Regarding results on quality of care, among all observed deliveries, the average pre-intervention quality score was 42.6% for the 20-item index and 53.9% for the 36-item index (Table 2). Deployment of the intervention increased the 20-item quality index by 3.1 percentage points (95% CI=−0.04,6.2) to 45.7%, and the 36-item index by 4.2 percentage points (95% CI=1.47,6.98) to 58.1%. Results for the subset of observations with some nighttime hours were slightly larger (4.1 and 4.7 percentage points for the 20-item and 36-item indices, respectively). Among all observations, delays in care were reduced by 11.24 minutes (95% CI=−16.47,−6.01), from an average of 74.3 minutes before the intervention to 63.1 minutes after the intervention. Similar results on delays in care were found for the subset of observations with some nighttime hours (−9.67 minutes [95% CI=−16.06, −3.29]). Estimates of the impact of the intervention on individual delay index items are shown in Supplement Table 7, with the largest effects on reducing time from arrival to first interaction and time from delivery to initiating breastfeeding.

**FIGURE 4:**
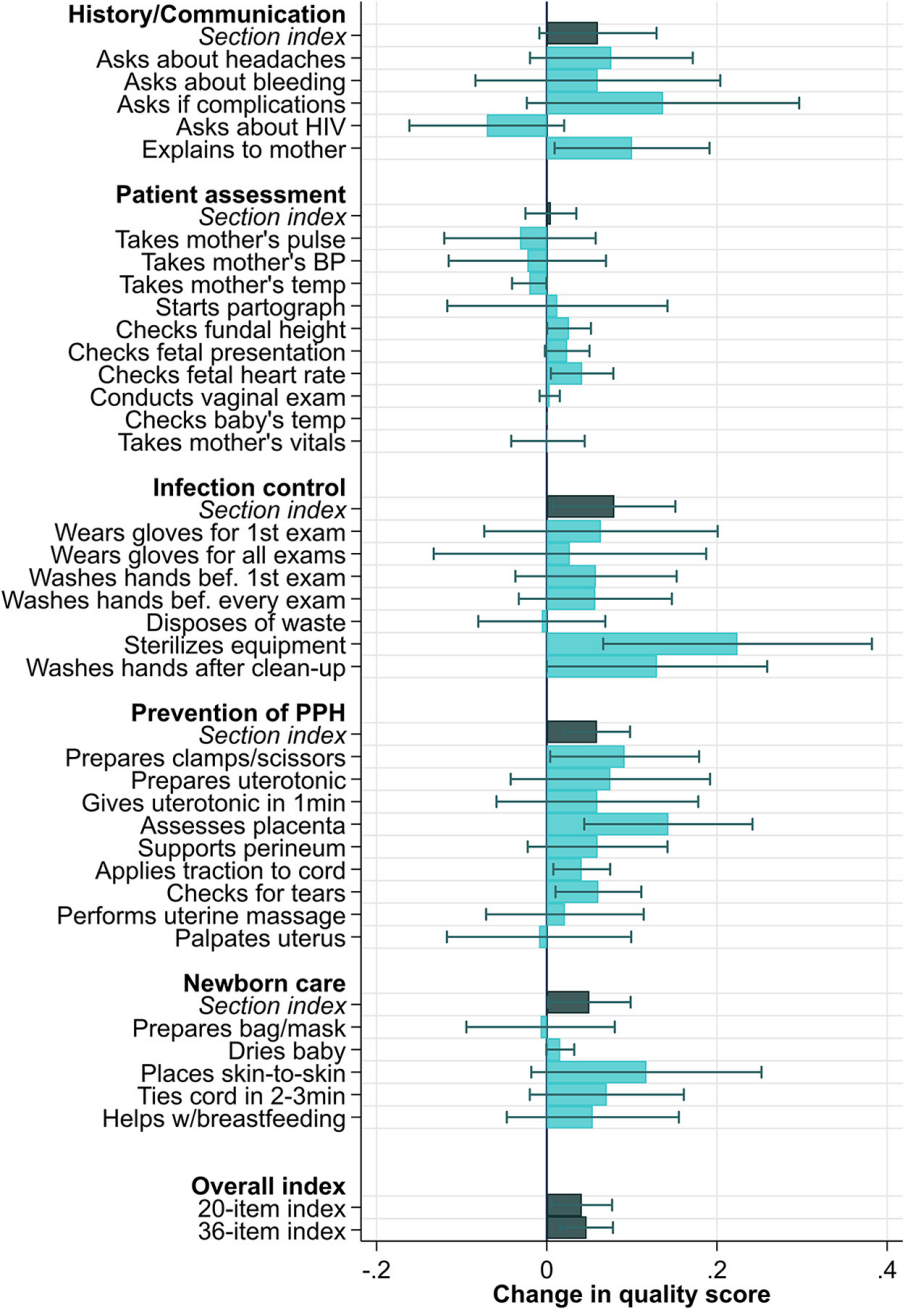
Impact of Solar Suitcase Intervention on Individual Quality Items, Section of Care Indices, and Overall Indices, for Observed Deliveries in Maternal Health Care Facilities in Uganda With Some Nighttime Hours^a^ ^a^ Results show point estimates and 95% confidence intervals. Standard errors are clustered at the facility level. Section and overall indices are the proportion of items performed out of the total section and overall total observed, respectively. Linear regression with facility fixed effects and clustered standard errors. Analysis includes only observed deliveries with some nighttime hours. Sterilizes equipment is coded as 1 for observations in which no reusable instruments were used (30% of observations). BP, blood pressure; PPH, postpartum hemorrhage. Analysis includes only observed deliveries with some nighttime hours (between 6:00 pm and 7:00 am).

Figure 4 shows the results of the impact of the intervention on individual items of the 20-item and 36-item indices and section indices. Point estimates and confidence intervals are shown in Supplement Table 8. The largest impacts of the intervention were found in infection control (8 percentage point increase [1 to 15]), prevention of postpartum hemorrhage (6 percentage point increase [2 to 10]), and newborn care (5 percentage point increase [0 to 10]). In terms of individual items, the intervention had the most significant impact on checking fetal heart rate, sterilization of equipment (usually conducted chemically with chlorine or chlorhexidine), preparing cord clamps for delivery, assessing completeness of the placenta, applying traction to the cord, checking for tears, and washing hands after clean-up. Health care workers also used the fetal Doppler that was included as part of the Solar Suitcase intervention. In nearly 40% of observed deliveries, health care workers used the Doppler to measure fetal heart rate after the intervention was deployed. While pairwise *P*-values indicated a significant impact of the intervention on individual items within these care domains, adjusting for multiple hypothesis testing within domains resulted in few significant results at the .05 level.

The largest impacts of the intervention were found in infection control, prevention of postpartum hemorrhage, and newborn care.

Sensitivity analyses produced similar results to the main analysis (Supplement Table 9–13).

### Maintenance

We examined the percentage of facilities with Solar Suitcase components in operation across 3 posttrial follow-up visits, up to 1 year after the completion of the trial. At 1 year after the end of the trial, 93% of facilities had the Solar Suitcase LED lights still in use (i.e., functioning and available). The percentage of facilities with the following components still in use 1 year after follow-up were fetal Doppler (90% of facilities), headlamps (83%), and USB ports (83%) (Supplement Table 14). The main challenges reported for why components were not in use were that they were malfunctioning, stolen, or missing and that newly arrived health care workers were not trained to use them. Additionally, 27% of facilities reported that LED lights from the Solar Suitcase, while functioning, did not last through the night or became dim.

## DISCUSSION

The results of this stepped-wedge cluster-randomized trial show that a solar energy system intervention in rural Ugandan maternity facilities was well adopted, increased brightness and adequacy of lighting for maternal and infant care, and led to increases in the quality of care received by women and newborns. While over 40% of deliveries were conducted by flashlight or kerosene lamp at baseline, receipt of the solar light intervention increased the proportion of deliveries conducted with adequate light to 100%. Deployment of the intervention also increased quality of care, particularly in the areas of infection control, prevention of postpartum hemorrhage, and newborn care, while decreasing delays in the provision of care.

Reports from health care workers confirmed the widespread use of the Solar Suitcase LED lights and USB ports. However, fewer used the associated devices: 56% of health care workers reported not making use of the headlamps and 30% reported not using the fetal Doppler. In interviews, a common explanation given from health care workers for the inconsistent use of the Doppler was insufficient gel to use with the device as well as technical difficulties in using it. These results are consistent with World Health Organization research finding that many complex medical devices in low-resource settings do not function as intended.^34^ Regarding maintenance measures, 93% of facilities reported using the LED lights 1 year after the end of the trial period. However, some challenges to maintained use were identified, with 27% of facilities reporting that the solar lights did not last through the night or became dim. In the long term, conducting an energy audit with facilities would produce valuable information on a facility's energy usage to tailor the Solar Suitcase or design and install other renewable solutions that meet facility needs.^35^

The introduction of reliable light decreased delays in performance of essential care actions. Delays in care, often referred to as the “third delay” in the 3-delays model of access to delivery care, can result in undiagnosed and untreated complications that increase the risk of maternal mortality and morbidity.^36^ This study found that adequate light can significantly reduce the time that passes between a patient's arrival at a facility and their first interaction with a health care provider, a time that is critically important for women with high-risk pregnancies who need to be triaged or transferred to a higher-level facility.^37^

We found that adequate light can significantly reduce the time that passes between a patient's arrival at a facility and their first interaction with a health care provider, which is critical for women with high-risk pregnancies.

Consistent with previous literature on maternal care quality in sub-Saharan Africa, the observed quality of maternity care in this sample of primary care Ugandan facilities was insufficient, with a large number of essential processes of care not provided.^38^^,^^39^ Before the intervention was deployed, providers were performing only 42% of the essential care items for safe deliveries. The introduction of adequate light increased this to 46%, including items of known clinical significance such as assessing the completeness of the placenta and checking for lacerations, a 9.5% increase. The improvement in quality observed in this study is similar to that of other quality improvement interventions in LMICs. An overview of systematic reviews found that the use of reminders to prompt providers to perform actions produced a median relative effect of 14%, while multifaceted interventions produced modest to moderate improvements in professional practice of between 5% and 20%.^40^

Overall, the results provide support for the importance of providing all Ugandan maternity facilities with reliable, bright light, but they also indicate that even when facilities have high-quality light, conditions may still be insufficient to ensure safe childbirth. These results underscore recommendations from global heath quality improvement committees that efforts to improve quality must include transformative, systemic changes across all levels of the health system.^41^^,^^42^

These results underscore global heath quality improvement committees' recommendations that efforts to improve quality must include transformative, systemic changes across all levels of the health system.

While the magnitude of the effect sizes on quality is modest, the intervention may have greater impacts in countries where access to reliable light is lower. Moreover, several potential benefits to improved lighting are not captured in this evaluation. For example, women may be more likely to decide to deliver in a well-lit facility and may also be less likely to be referred to higher-level facilities due to electricity interruptions, which can be dangerous when such facilities are distant and transportation is unreliable. There may also be impacts on health care worker morale and retention, which we will examine in future publications. Overall, given limited resources in LMICs, cost-effectiveness analyses could help clarify priorities for health sector investment in health system strengthening across domains of energy access, medical equipment and supplies, health financing, service delivery, and human resources.^43^

This study has several strengths. We used direct clinical observations of care, which is the gold standard in assessing quality of care. We also used both observations of sources and brightness of light and light sensors to validate our results. Results from the light sensors found that the intervention increased the number of daily minutes of light and the brightness of the room during nighttime hours, corroborating the enumerator-reported results of brightness. Our study design, a stepped-wedge cluster-randomized controlled trial, allowed for observation before and after deployment of the intervention in all facilities and minimized risk of confounders.

### Limitations

This study also has several limitations. First, while direct observation of care is the gold standard in assessing quality, it also has limitations including the possibility that providers change their behavior as a result of being watched (the Hawthorne effect). The possibility also exists that enumerators do not always score the care they observe accurately. In our sensitivity analyses, we did not find strong evidence for either of these effects on the results. Second, the lack of blinding is a limitation. Enumerators may have scored quality of care better when a facility received a Solar Suitcase because they anticipated a positive effect of the intervention. However, the quality metric we used is validated as a reliable, objective measure. Moreover, enumerators were not deployed to the same facilities in all 3 periods. Another possibility is that enumerators record higher or lower scores when there is better light because they can see more clearly. However, the direction of this effect is ambiguous, and we found no evidence of this in our qualitative debriefings with enumerators. Finally, our analysis focused on uncomplicated vaginal deliveries and was not designed or powered for analysis of quality of care during complications. Thus, these results do not speak to any potential impact of better lighting on the management of maternal or newborn complications.

## CONCLUSIONS

Universal access to modern energy sources and safe childbirth are both key sustainable development goals.^44^ Moreover, identifying effective approaches to improving the quality of health care in LMICs is an urgent public health goal.^45^ We find that reliable light is an important driver of timely and adequate health care and may improve providers' ability and timeliness in performing actions needed to reduce the risk of postpartum hemorrhage. Investment in modern and renewable energy systems for health care facilities is a critical priority; our results support recommendations by international organizations such as SEforAll, USAID, and the World Bank to develop guidance on energy access and clinical equipment needs of community-level primary health facilities and to facilitate large-scale facility electrification efforts.^12^^,^^46^^,^^47^ However, quality of care may remain low even in the presence of reliable light without a broader, systemic approach to high-quality health systems strengthening.

## Supplementary Material

GHSP-D-21-00205-Supplement.pdf

## Notes

**Peer Reviewed**

**First published online:** December 9, 2021.
