# Supplementary material for: Impact of Solar Light and Electricity on the Quality and Timeliness of Maternity Care: A Stepped-Wedge Cluster-Randomized Trial in Uganda
Source: Glob Health Sci Pract. 2021 Dec 31;9(4):777–92. doi: 10.9745/GHSP-D-21-00205 (PMC8691890; doi:10.9745/GHSP-D-21-00205)
Supplement: GHSP-D-21-00205-Supplement.pdf [file GHSP-D-21-00205-Supplement.pdf]

Supplement to: Rokicki S, Mwesigwa B, Waiswa P, Cohen J. Impact of solar light and electricity on the quality and timeliness of maternity care: a stepped-wedge cluster-randomized trial in Uganda. *Glob Health Sci Pract*. 2021;9(4). https://doi.org/10.9745/GHSP-D-21-00205

Supplement

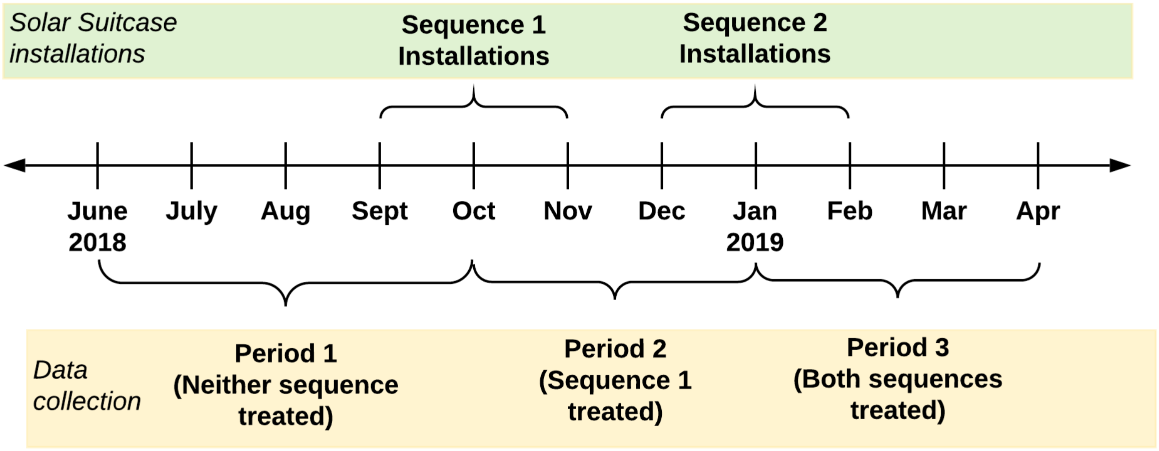

Supplement Figure 1. Timeline of data collection and Solar Suitcase installation

Notes: After installation, there was an adjustment period of 4-6 weeks in which no observations were conducted to allow health workers to become accustomed to using the Solar Suitcase. Installations were conducted by a local solar contracting firm based in Uganda trained and managed by We Care Solar. One Solar Suitcase was installed in each facility, with 2-4 overhead LED lights for each delivery room, depending on its size, and all facilities received a total of 4 LED lights. Most facilities in the sample had only one delivery room. After installation, the solar contracting firm technician provided a lesson on how to use the Solar Suitcase and its components to all maternity health workers and the medical officer in-charge. If any health workers were not present, the technician coordinated with the facility to return two to four weeks later to conduct another lesson. The technician also conducted an in-person meeting two weeks after installation and phone call follow-up after four to six weeks to ensure the Solar Suitcase was functioning and the health workers were using all components. The technician also followed up with maintenance requests throughout the remainder of the study.

Supplement to: Rokicki S, Mwesigwa B, Waiswa P, Cohen J. Impact of solar light and electricity on the quality and timeliness of maternity care: a stepped-wedge cluster-randomized trial in Uganda. *Glob Health Sci Pract*. 2021;9(4). https://doi.org/10.9745/GHSP-D-21-00205

www.wecaresolar.org

We Care Solar Suitcase

Version 2 Product Specifications

Solar Suitcase

WCS-200-(v2)

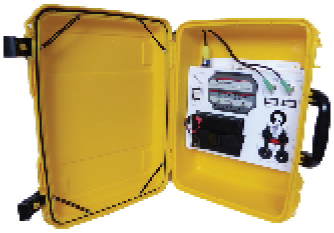

Advanced solar controller, ports and plugs, in watertight and dust-proof high-quality suitcase, made to hold up in harsh environments. System contains an integrated 12V, 12.8Ah battery, which is charged by a solar panel. Suitcase has 2 WCS Lighting Connectors, two 12VDC Accessory (lighter) Sockets, and two Expansion Ports to allow for optional accessories. A homerun cable and installation hardware are included.

Size and Weight

Height:	**19.75** inches (501.6 mm)
Width:	**15.53** inches (394.4 mm)
Depth:	**7.48** inches (190 mm)
Weight:	**30** pounds (13.6 kg)

Power

Max. Power:	**180** watts
Max. Current:	**15** amps
Operating Voltage:	**11.4** to **14.4** volts
Operating Temp.:	**0**°C to **45**° C

Hi-Efficiency Lights

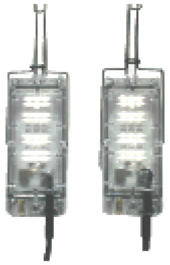

Four high-efficiency, rugged, 4W LED lights provide over 325 lumens each and have a color-rendering capacity optimized for medical use. The lights connect to the suitcase via the WCS Lighting Connectors. They run directly on12VDC and are designed by WCS to withstand exposure to heat, moisture and dirt.

Size and Weight

Height:	**5** inches (127 mm)
Width:	**2.2** inches (56 mm)
Depth:	**0.9** inch (23 mm)
Cord:	**33** feet (10m)

Power

Load:	**4** watts
CRI:	>85 (Ra)
Color Temperature:	**5000**° K
Life Expectancy:	**50,000** hours

Phone Charger

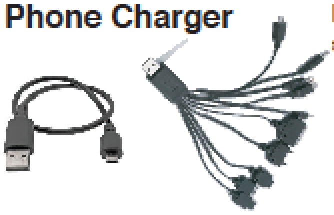

Both a USB > microUSB cable, and a 10-in-1 USB cable are supplied, supporting charging of a wide variety of cell phones.

Headlamps

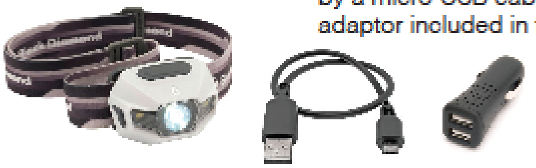

Adjustable headlamps are equipped with LEDs and are charged by a micro-USB cable. The micro-USB cable plugs into a 12V USB adaptor included in the Solar Suitcase.

Supplement to: Rokicki S, Mwesigwa B, Waiswa P, Cohen J. Impact of solar light and electricity on the quality and timeliness of maternity care: a stepped-wedge cluster-randomized trial in Uganda. *Glob Health Sci Pract*. 2021;9(4). https://doi.org/10.9745/GHSP-D-21-00205

Supplement Figure 2. Solar Suitcase product specification and technical details (Version 2 of Suitcase, used in the study)

Supplement to: Rokicki S, Mwesigwa B, Waiswa P, Cohen J. Impact of solar light and electricity on the quality and timeliness of maternity care: a stepped-wedge cluster-randomized trial in Uganda. *Glob Health Sci Pract*. 2021;9(4). https://doi.org/10.9745/GHSP-D-21-00205

We Care Solar Suitcase

2020 Product Specifications

www.wecaresolar.org

Solar Suitcase

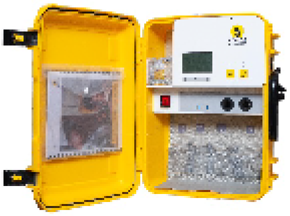

Advanced solar controller, sockets, controls, and battery – all in a water-and-dust tight suitcase, made to hold up in harsh environments. System contains an integrated 12V, 20Ah battery, which is charged by a solar panel. Suitcase has two light sockets, two 12VDC accessory (lighter) sockets, two USB ports, and two expansion ports to allow for optional accessories or additional lights. A 40' homerun cable and all necessary installation hardware is included. Custom display and user-interface make operation simple and intuitive.

Size and Weight

Height:	19.75 inches (501.6 mm)
Width:	15.53 inches (394.4 mm)
Depth:	7.48 inches (190 mm)
Weight:	32 pounds (14.5 kg)

Power

Max. Power:	240 watts
Max. Current:	20 amps
Operating Voltage:	11.4 to 14.4 volts
Operating Temp.:	0° C to 40° C

LED Lights

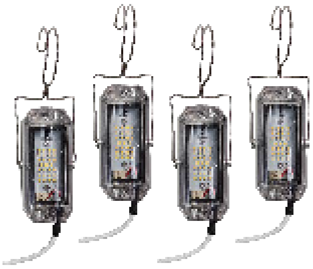

Four high-efficiency, rugged, LED lights provide up to 720 lumens and have a color-rendering capacity suitable for medical use. Two lights connect to the Solar Suitcase via the integrated light sockets and two connect through the Light Expansion Box. They are designed by We Care Solar to withstand exposure to heat, moisture, dirt and impact.

Size and Weight

Height:	4.5 inches (11 cm)
Width:	2 inches (5 cm)
Depth:	1 inch (2.5 cm)
Cord:	33 feet (10 m)
Weight:	1.1 pounds (0.5 kg)

Power

Load:	1-6 watts (dimmable)
CRI:	>91 (R9>70)
Color Temp:	4950 K

Battery

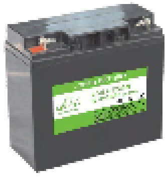

One 20Ah Lithium Ferrous Phosphate (LFP) battery provides lightweight and rugged energy storage.

Size and Weight

Height:	6.6 inches (167 mm)
Width:	3 inches (77 mm)
Length:	7.2 inches (181.5 mm)
Weight:	6.7 pounds (3.0 kg)

Power

Max Capacity:	256 watt-hours
Max Usable Capacity:	256 watt-hours
Life Expectancy:	4-7 years

Light Expansion Box

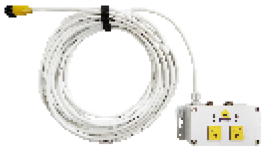

The Light Expansion Box provides switches and sockets that can be installed in a room up to 40' (12.1m) from the Solar Suitcase. The box supports two additional lights and connects to the Solar Suitcase via the expansion socket located on the Solar Suitcase control panel.

Weight

Weight: 1.7 pounds (.77 kg)

We Care Solar **2150 Allston Way, Suite 340, Berkeley, CA 94704, U.S.A. info@wecaresolar.org**

Supplement Figure 3. Solar Suitcase product specification and technical details (Version 3 of Suitcase)

Supplement to: Rokicki S, Mwesigwa B, Waiswa P, Cohen J. Impact of solar light and electricity on the quality and timeliness of maternity care: a stepped-wedge cluster-randomized trial in Uganda. *Glob Health Sci Pract*. 2021;9(4). https://doi.org/10.9745/GHSP-D-21-00205

Supplement Table 1. Inclusion and exclusion criteria for participants and enumerator minimum qualifications

Category	Initial screening	Updated eligibility
Facilities	i) level II, III, or IV;<br>ii) open 24 hours a day;<br>iii) had unreliable overhead light (defined as zero or one functioning sources of overhead light in the maternity ward, which was interrupted at least a few times a week);<br>iv) did not automatically refer women out during blackouts; and<br>v) the medical officer in-charge was willing to participate.	Enumerators began observing deliveries in facilities that met initial screen criteria; if overhead light was determined to be reliable by the enumerator or the facility had inadequate frequency of deliveries (<10/month), the facility was excluded from the sample.
Health providers	i) worked in the maternity ward<br>ii) willing to participate	
Mothers	Inclusion:<br>i) pregnant<br>ii) aged 16 and over<br>iii) admitted to facilities for labor and delivery<br>iv) provided written informed consent<br><br>Exclusion:<br>i) pregnant women presenting for conditions other than normal labor and delivery<br>ii) women who were immediately transferred to another hospital<br>iii) women who delivered outside of the maternity ward<br>iv) women for whom the health worker objected to the observation.	
Enumerators	i) Minimum of a diploma or certificate in Comprehensive Nursing or Midwifery from an accredited Uganda National Council of Higher Education<br>ii) Trained for approximately two weeks on research protocol, data collection methods, and human subjects research	

Supplement to: Rokicki S, Mwesigwa B, Waiswa P, Cohen J. Impact of solar light and electricity on the quality and timeliness of maternity care: a stepped-wedge cluster-randomized trial in Uganda. *Glob Health Sci Pract*. 2021;9(4). https://doi.org/10.9745/GHSP-D-21-00205

Supplement Table 2. Details on study procedures

	**Procedure**
**Randomization**	30 facilities were randomized to receive the intervention in one of two sequences, with 15 facilities in each sequence (step). Randomization was conducted using Stata's *randomize* stratifying by geographic cluster and average adequate light during period 1 (above or below the median for all facilities), and balancing on average quality of care at period 1 and average facility volume over the three months previous to the start of period 1.
**Enumerator protocol**	To reduce disruption and influence, enumerators were trained to avoid interaction with providers and patients. If an enumerator was uncertain about whether a checklist item was conducted by the health worker, they were instructed to indicate "don't know". One or two enumerators remained at facilities for a minimum of two weeks, and until they had recorded a minimum of 3 observations in the daytime and 3 observations in which at least some part of the observation occurred during the nighttime hours of 6pm to 7am. On average enumerators remained at facilities for 3 to 6 weeks per observation period and observed all or nearly all deliveries that occurred during that time. Enumerators were provided with digital watches for recording timestamps.
**Enumerator inter-rater reliability**	To maximize inter-rater reliability, enumerators were extensively trained on the definition of each item in the observation checklist under the leadership of the study obstetrician. Enumerators practiced conducting clinical observations in groups at a local hospital prior to study start, followed by debriefs to identify, discuss, and resolve discrepancies in enumerator responses. Refresher trainings and debriefs occurred biweekly in-person throughout the study period. Enumerators also trained and agreed on the definition for brightness of light using photographs.

Supplement to: Rokicki S, Mwesigwa B, Waiswa P, Cohen J. Impact of solar light and electricity on the quality and timeliness of maternity care: a stepped-wedge cluster-randomized trial in Uganda. *Glob Health Sci Pract*. 2021;9(4). https://doi.org/10.9745/GHSP-D-21-00205

Supplement Table 3. Primary and secondary outcomes measurements and definitions

Outcome	Measurement	Definition
Bright throughout labor and delivery	Reported by enumerator during each of 4 sections of observation as "very bright", "somewhat bright", "dim", or "pitch black"<br><br>Definitions of categories of brightness:<br><br>"Very bright" indicates that the provider is able to see the mother well to conduct all procedures.<br><br>"Somewhat bright" indicates that the provider is able to see the mother adequately to conduct most procedures but would strain to read or see details.<br><br>"Dim" indicates that it is difficult for the provider to see the mother well to conduct procedures, and is unable to read or see details.<br><br>"Pitch black" indicates that there is no light in the room	Equal to 1 if responses to all 4 sections is "very bright" or "bright".<br><br>Equal to 0 otherwise
Satisfactory light source used throughout labor and delivery	Reported by enumerator during each of 4 sections of observation as "Daylight", "Solar Suitcase", "Grid", "Overhead Solar", "Generator", "Kerosene lamp", "Solar lamp", "Candle", "Torch", or "Other"	Equal to 1 if responses to all 4 sections is "Daylight", "Solar Suitcase", "Grid", "Overhead Solar", or "Generator"<br><br>Equal to 0 otherwise
Adequate light throughout labor and delivery	Reported by enumerator; combination of brightness and satisfactory light source	Equal to 1 if responses to all 4 sections is both bright (as defined above) and from satisfactory source (as defined above)<br><br>Equal to 0 otherwise
Number of minutes of light in a 24-hour day	Sensor	A maximum sensor value is calculated for each facility;<br><br>Variable is equal to the number of minutes over a threshold of 20% of the maximum
Average brightness during daytime hours (7am to 6pm)	Sensor	A maximum sensor value is calculated for each facility, and brightness at each hour is calculated as the percentage of the maximum.<br><br>Average brightness during daytime hours is equal to the average percentage across the hours of 7am to 6pm

Average brightness during nighttime hours (6pm to 7am)	Sensor	A maximum sensor value is calculated for each facility, then brightness at each hour is calculated as the percentage of the maximum.<br><br>Average brightness during nighttime hours is equal to the average percentage across the hours of 6pm to 7am
20-item quality of care index	Enumerator report using checklist tool	Percentage of total items completed (individual items shown in Supplement Table 2). Missing items are ignored.
36-item quality of care index	Enumerator report using checklist tool	Percentage of total items completed (individual items shown in Supplement Table 2). Missing items are ignored.
6-item delay index	Enumerator report using timestamps on checklist tool	Sum of number of minutes of delay across the following 6 measures of time between: (1) facility arrival and first contact with health care worker, (2) facility arrival and first examination, (3) delivery and provision of uterotonic, (4) delivery and assessment of perineal and vaginal lacerations, (5) delivery and drying baby with towel, and (6) delivery and initiation of breastfeeding.<br><br>Measure is set to missing if any of the components of the index are missing.

Supplement to: Rokicki S, Mwesigwa B, Waiswa P, Cohen J. Impact of solar light and electricity on the quality and timeliness of maternity care: a stepped-wedge cluster-randomized trial in Uganda. *Glob Health Sci Pract*. 2021;9(4). https://doi.org/10.9745/GHSP-D-21-00205

Supplement Table 4. Individual quality items and proportion of observations in which item was performed before and after deployment of intervention

Outcome	Before intervention (%)	After intervention (%)
**History taking/communication**	55%	60%
**Asks whether she has experienced headaches and/or blurred vision during the current pregnancy.	12%	20%
**Asks whether she has experienced vaginal bleeding during the current pregnancy.	36%	39%
Asks about complications during <u>previous</u> pregnancies.	51%	65%
**Offers woman an HIV test **or** checks her HIV status.	93%	85%
**At least once, explains what will happen in labour to woman and/or her support person.	83%	91%
**Patient assessment**	44%	45%
**Takes pulse at initial exam.	12%	10%
**Takes blood pressure at initial exam.	16%	13%
Takes mother's temperature at initial exam.	2%	0%
**Starts partograph to monitor progress of labour.	19%	21%
Checks fundal height.	96%	99%
Checks fetal presentation by palpation of abdomen	97%	99%
Checks fetal heart rate with fetoscope/Doppler/ultrasound	93%	98%
Performs vaginal examination.	100%	100%
Checks baby's temperature after delivery.	0%	0%
**Takes mother's vital signs after delivery.	4%	3%
**Infection control**	55%	65%
**Wears sterile gloves for initial vaginal examination.	76%	84%
Wears sterile surgical gloves for all vaginal examinations.	67%	74%
Washes his/her hands with soap and water or uses alcohol hand rub before initial examination.	12%	22%
**Washes his/her hands with soap and water or uses alcohol hand rub prior to every examination of woman.	9%	18%
Disposes of all contaminated waste in leak-proof containers	93%	94%
Sterilizes or uses high-level disinfection for all reusable instruments	60%	81%

Supplement to: Rokicki S, Mwesigwa B, Waiswa P, Cohen J. Impact of solar light and electricity on the quality and timeliness of maternity care: a stepped-wedge cluster-randomized trial in Uganda. *Glob Health Sci Pract*. 2021;9(4). https://doi.org/10.9745/GHSP-D-21-00205

Washes his/her hands with soap and water or uses alcohol hand rub after cleaning up.	69%	88%
**Prevention of PPH**	57%	62%
Prepares disposable cord ties/clamps AND sterile scissors/blade.	84%	91%
**Prepares uterotonic for active management of third stage of labor.	57%	64%
**Administers correct dose of uterotonic within 1 minute of delivery.	28%	29%
**Assesses completeness of the placenta and membranes.	26%	36%
As baby's head is delivered, supports perineum.	86%	92%
Applies traction to the cord while applying suprapubic counter traction.	94%	96%
**Assesses for perineal and vaginal lacerations.	8%	14%
Performs uterine massage immediately following the delivery of the placentae	86%	86%
**Palpates uterus after delivery of placenta.	57%	54%
**Newborn care**	60%	66%
**Prepares self-inflating ventilation bag (250 or 500 mL), newborn face mask size 0, and face mask size 1.	13%	14%
**Immediately dries baby with towel.	98%	100%
**Places baby on mother's abdomen or chest skin-to-skin.	69%	81%
**Ties or clamps cord after pulsations stop, or 2-3 min after birth.	57%	65%
**Initiates breastfeeding within the first hour after birth.	76%	81%
**20-item index	42%	46%
36-item index	54%	58%

Notes: 20-item index is composed of items prefixed with **. 36-item index includes all items.

Supplement to: Rokicki S, Mwesigwa B, Waiswa P, Cohen J. Impact of solar light and electricity on the quality and timeliness of maternity care: a stepped-wedge cluster-randomized trial in Uganda. *Glob Health Sci Pract*. 2021;9(4). https://doi.org/10.9745/GHSP-D-21-00205

Supplement Table 5. Sensitivity analyses

Category	Sensitivity analyses
Alternative specifications	• For continuous outcomes, multilevel regression models with facility random effects, adjusting for randomization blocking variables and facility characteristics in addition to duration of time in facilities<br>• For dichotomous outcomes, we conducted logistic regression models with facility fixed effects.
Adjustment of standard errors	We also compared our results to those using a wild cluster bootstrap method, which is advised when the number of clusters is less than 50.
Missing data	Missing data occurred for several reasons including: the patient was referred to another facility before delivering, the enumerator was not present or able to conduct some part of the observation, or the enumerator was not sure whether an item was performed or not and indicated 'don't know' on the checklist. For the main analysis, when observations included missing items, the index was calculated as the total items performed over the total observed by the enumerator.<br>In sensitivity analyses, we conducted:<br>(1) a complete-case analysis<br>(2) multiple imputation of missing data using an iterative Markov chain Monte Carlo method assuming an underlying multivariate normal model on 10 imputed datasets.
Enumerator bias or provider bias due to Hawthorne effects of being observed	• Enumerator bias may occur if the way enumerators marked responses changed over time; this could be especially prominent in the first days of observation, before enumerators solidify their skills. As a sensitivity analysis, we dropped the first five observations of an enumerator to reduce this potential scoring bias at the start of the study.<br>• Additionally, Hawthorne effects would occur if a provider changed their behaviour because they knew they were being observed. Since some evidence suggests that Hawthorne effects fade over time we conducted a sensitivity analysis in which we dropped the first five observations of a provider. Because a delivery may have more than one provider providing care (either simultaneously or sequentially over time as one provider ends their shift and another provider takes over), we assigned the delivery observation to the provider who was present throughout the entire observation. 79% of observations had only one provider present throughout the entire observation. For the 21% of observations with more than one provider present throughout the observation, we assigned the observation to the provider who provided the most care (i.e. the highest number of items on the checklist).
Check on selection of patients or providers into better lit facilities	We tested if patient- or provider-level characteristics changed before and after installation of the intervention; a change could indicate a selection of providers or patients into better lit facilities.
Inclusion of observations that resulted in multiple	We included the 15 observations that resulted in a multiple birth (n=8), stillbirth (n=5), and infant death (n=2) that were dropped from the main analysis.

Supplement to: Rokicki S, Mwesigwa B, Waiswa P, Cohen J. Impact of solar light and electricity on the quality and timeliness of maternity care: a stepped-wedge cluster-randomized trial in Uganda. *Glob Health Sci Pract*. 2021;9(4). https://doi.org/10.9745/GHSP-D-21-00205

births, stillbirth, or infant death	

Supplement Table 6. Health worker's self-reported use of Solar Suitcase components (n=87)

	Always (Every Delivery)	Often (Most Deliveries)	Sometimes (Some Deliveries)	Rarely (Few Deliveries)	Never
Use of lights during the day	4 (5%)	3 (3%)	1 (1%)	19 (22%)	53 (61%)
Use of lights during the night	71 (82%)	6 (7%)	2 (2%)	3 (3%)	5 (6%)
Use of headlamps	8 (9%)	8 (9%)	17 (20%)	21 (24%)	28 (32%)
Use of fetal doppler	20 (23%)	16 (18%)	22 (25%)	16 (18%)	10 (11%)
USB port to charge phone	15 (17%)	28 (32%)	11 (13%)	9 (10%)	21 (24%)

Supplement to: Rokicki S, Mwesigwa B, Waiswa P, Cohen J. Impact of solar light and electricity on the quality and timeliness of maternity care: a stepped-wedge cluster-randomized trial in Uganda. *Glob Health Sci Pract*. 2021;9(4). https://doi.org/10.9745/GHSP-D-21-00205

Supplement Table 7. Impact of intervention on individual delay index items

	**Arrival to 1st interaction**	**Arrival to initial exam**	**Delivery to adminis-tration of uterotonic**	**Delivery to checking for tears**	**Delivery to drying baby**	**Delivery to breast-feeding**	**Delay index**
**Panel A: All observed deliveries**							
Treated	-5.61 (-10.83, -0.38)	-4.4 (-12.57, 3.77)	-1.01 (-3.20, 1.18)	-1.12 (-2.02, -0.21)	-0.40 (-0.68, -0.12)	-4.34 (-7.85, -0.83)	-11.24 (-16.47, -6.01)
Constant	13.93 (11.85, 16.01)	20.65 (17.37, 23.92)	5.36 (4.48, 6.24)	9.68 (9.31, 10.06)	0.92 (0.80, 1.03)	36.18 (34.78, 37.58)	74.33 (72.15, 76.52)
Observations							
**Panel B: Observed deliveries with some nighttime hours**							
Treated	-5.77 (-13.25, 1.70)	-2.23 (-13.59, 9.14)	-0.92 (-3.76, 1.91)	-0.95 (-2.04, 0.13)	-0.37 (-0.70, -0.05)	-4.69 (-8.78, -0.60)	-9.67 (-16.06, -3.29)
Constant	14.87 (12.20, 17.53)	21.25 (17.19, 25.30)	5.46 (4.44, 6.48)	9.77 (9.37, 10.17)	0.93 (0.81, 1.04)	36.13 (34.65, 37.61)	75.97 (73.60, 78.35)
Observations	739	736	644	670	731	741	575

Notes: Results show point estimate and 95% confidence interval. Standard errors are clustered at facility level. Nightime hours refer to hours between 6pm to 7pm. Breastfeeding delay measured only among deliveries in which breastfeeding occurred within an hour.

Supplement to: Rokicki S, Mwesigwa B, Waiswa P, Cohen J. Impact of solar light and electricity on the quality and timeliness of maternity care: a stepped-wedge cluster-randomized trial in Uganda. *Glob Health Sci Pract*. 2021;9(4). https://doi.org/10.9745/GHSP-D-21-00205

Supplement Table 8. Results of impact of intervention on individual quality items, with native p-values and p-values adjusted for multiple hypothesis testing

	n	Point estimate	Confidence interval	Naïve p-value	Adjusted p-value[a]
**History/Communication**					
Section index	743	0.06	(-0.01,0.13)	0.084	
Asks about headaches	735	0.08	(-0.02,0.17)	0.115	0.152
Asks about bleeding	739	0.06	(-0.08,0.20)	0.406	0.406
Asks if complications	737	0.14	(-0.02,0.30)	0.088	0.152
Asks about HIV	739	-0.07	(-0.16,0.02)	0.121	0.152
Explain to mother	724	0.1	(0.01,0.19)	0.034	0.152
**Patient assessment**					
Section index	743	0	(-0.03,0.03)	0.745	
Takes mother's pulse	739	-0.03	(-0.12,0.06)	0.452	0.763
Takes mother's BP	739	-0.02	(-0.12,0.07)	0.593	0.763
Takes mother's temperature	739	-0.02	(-0.04,-0.00)	0.046	0.138
Starts partograph	724	0.01	(-0.12,0.14)	0.875	0.948
Checks fundal height	739	0.03	(0.00,0.05)	0.046	0.138
Checks fetal presentation	739	0.02	(-0.00,0.05)	0.071	0.16
Checks fetal heart rate	739	0.04	(0.00,0.08)	0.027	0.138
Conducts vaginal exam	739	0	(-0.01,0.02)	0.547	0.763
Checks baby's temp	742	0	(0.00,0.00)	.	
Takes mother's vitals	741	0	(-0.04,0.04)	0.948	0.948
**Infection Control**					
Section index	743	0.08	(0.01,0.15)	0.080	
Wears gloves for 1st exam	739	0.06	(-0.07,0.20)	0.359	0.503
Wears gloves for all exams	723	0.03	(-0.13,0.19)	0.776	0.869
Washes hands bef. 1st exam	739	0.06	(-0.04,0.15)	0.237	0.415
Washes hands bef. Every exam	723	0.06	(-0.03,0.15)	0.22	0.415
Disposes of waste	742	-0.01	(-0.08,0.07)	0.869	0.869
Sterilizes equipment	742	0.22	(0.07,0.38)	0.007	0.049
Washes hands after clean-up	742	0.13	(-0.00,0.26)	0.052	0.182
**Prevention of PPH**					
Section index	743	0.06	(0.02,0.10)	0.004	
Prepares clamps/scissors	724	0.09	(0.00,0.18)	0.043	0.097
Prepares uterotonic	724	0.07	(-0.04,0.19)	0.196	0.294
Gives uterotonic in 1min	722	0.06	(-0.06,0.18)	0.321	0.413
Assesses placenta	741	0.14	(0.04,0.24)	0.007	0.06

Supports perineum	742	0.06	(-0.02,0.14)	0.136	0.245
Applies traction to cord	741	0.04	(0.01,0.07)	0.018	0.06
Checks for tears	741	0.06	(0.01,0.11)	0.02	0.06
Performs uterine massage	741	0.02	(-0.07,0.11)	0.62	0.698
Palpates uterus	742	-0.01	(-0.12,0.10)	0.838	0.838
**Newborn care**					
Section index	743	0.05	(0.00,0.10)	0.044	
Prepares bag/mask	724	-0.01	(-0.09,0.08)	0.867	0.867
Dries baby	742	0.02	(-0.00,0.03)	0.03	0.15
Places skin-to-skin	742	0.12	(-0.02,0.25)	0.098	0.187
Ties cord in 2-3min	742	0.07	(-0.02,0.16)	0.112	0.187
Helps with breastfeeding	742	0.05	(-0.05,0.16)	0.25	0.313
**Quality indices**					
20-item index	743	0.04	(0.01,0.08)	0.024	
36-item index	743	0.05	(0.02,0.08)	0.004	

Notes: [a]Multiple hypothesis testing conducted on individual items within each section using an iterative Markov chain Monte Carlo method assuming an underlying multivariate normal model on 10 imputed datasets.

Supplement Table 9. Results on primary and secondary outcomes using linear regression random effects model for all observed deliveries (panel A) and for observed deliveries with some nighttime hours (panel B)

	Deliveries with bright light (%)	Deliveries with satisfactory light source (%)	Deliveries with adequate light (%)	20-item quality index (%)	36-item quality index (%)	6-item delays index (%)
**Panel A: All observed deliveries**						
Treated	44.1	34.0	45.3	3.15	4.25	-10.64
	(36.6, 51.5)	(28.4, 39.7)	(38.0, 52.6)	(0.09, 6.22)	(1.56, 6.93)	(-15.75, -5.52)
Constant	-15.3	79.9	-8.3	55.29	57.33	156.63
	(-77.2, 46.6)	(2.5, 157.3)	(-63.6, 46.9)	(20.67, 89.91)	(30.91, 83.74)	(36.46, 276.80)
Observations	1118	1118	1118	1118	1118	805
**Panel B: Observed deliveries with some nighttime hours**						
Treated	57.8	45.1	59.3	4.35	4.90	-9.19
	(46.6, 68.9)	(36.2, 53.9)	(48.3, 70.3)	(0.86, 7.84)	(1.90, 7.91)	(-15.42, -2.96)
Constant	-7.7	118.7	-5.4	61.43	62.92	181.22
	(-124.3, 108.9)	(-35.4, 272.8)	(-113.4, 102.6)	(26.66, 96.20)	(37.10, 88.74)	(45.73, 316.70)
Observations	743	743	743	743	743	575

Notes: Results show point estimate and 95% confidence interval. "Constant" indicates the mean outcome in facilities without the intervention. "Treated" indicates the change in average outcome due to the intervention. Standard errors are clustered at facility level. Nightime hours refer to hours between 6pm to 7pm. Outcomes with (%) expressed as percentage points. Bright light indicates enumerator reported "perfectly bright" or "bright" (as opposed to "dim" or "dark") across all four sections of the observation. Satisfactory light source indicates that the light source during all four sections was either daylight, the grid, solar, or a generator. Adequate light indicates that all four sections of the observations used a satisfactory light source and were reported to be bright. Quality of care indices are defined in Supplement Tables 1 and 2. Delays index is missing for observations in which any one of the 6 delays items is missing.

Supplement to: Rokicki S, Mwesigwa B, Waiswa P, Cohen J. Impact of solar light and electricity on the quality and timeliness of maternity care: a stepped-wedge cluster-randomized trial in Uganda. *Glob Health Sci Pract*. 2021;9(4). https://doi.org/10.9745/GHSP-D-21-00205

Supplement Table 10: Logistic fixed effects regression models of impact of intervention on measures of light

	All observed deliveries			Observed deliveries with some nighttime hours		
	**Bright light OR**	**Satisfactory light source OR**	**Adequate light OR**	**Bright light OR**	**Satisfactory light source OR**	**Adequate light OR**
Treated	69.057 (29.974, 159.098)	48.662 (19.626, 120.652)	54.537 (26.251, 113.302)	172.532 (58.716, 506.965)	179.703 (41.988, 769.107)	182.266 (61.966, 536.114)
Observations	1118	1118	1118	743	704	743

Notes: OR=odds ratio. Results show odds ratio and 95% confidence interval for the facilities with the intervention compared to facilities without. Nightime hours refer to hours between 6pm to 7pm. Standard errors clustered at facility level. Bright light indicates enumerator reported “perfectly bright” or “bright” (as opposed to “dim” or “dark”) across all four sections of the observation. Satisfactory light source indicates that the light source during all four sections was either daylight, the grid, solar, or a generator. Adequate light indicates that all four sections of the observations used a satisfactory light source and were reported to be bright.

Supplement to: Rokicki S, Mwesigwa B, Waiswa P, Cohen J. Impact of solar light and electricity on the quality and timeliness of maternity care: a stepped-wedge cluster-randomized trial in Uganda. *Glob Health Sci Pract*. 2021;9(4). https://doi.org/10.9745/GHSP-D-21-00205

Supplement Table 11. Comparison of p-values using clustering and wild bootstrap

	**Deliveries with bright light (%)**	**Deliveries with satisfactory light source (%)**	**Deliveries with adequate light (%)**	**20-item quality index (%)**	**36-item quality index (%)**	**6-item delays index (%)**
**Panel A: All observed deliveries**						
Treated (point estimate)	43.7	33.3	45.0	3.10	4.23	-11.24
p-value from clustering	<0.001	<0.001	<0.001	0.052	0.004	<0.001
p-value from wild cluster bootstrap	<0.001	<0.001	<0.001	0.06	0.007	0.001
**Panel B: Observed deliveries with some nighttime hours**						
Treated (point estimate)	57.5	44.7	59.0	4.12	4.68	-9.67
p-value from clustering	<0.001	<0.001	<0.001	0.024	0.004	0.004
p-value from wild cluster bootstrap	<0.001	<0.001	<0.001	0.03	0.005	0.013

Notes: Wild bootstrap using null imposed, 1000 replications, and Rademacher weights using the boottest command in Stata 15. Bright light indicates enumerator reported “perfectly bright” or “bright” (as opposed to “dim” or “dark”) across all four sections of the observation. Satisfactory light source indicates that the light source during all four sections was either daylight, the grid, solar, or a generator. Adequate light indicates that all four sections of the observations used a satisfactory light source and were reported to be bright. Quality of care indices are defined in Supplement Tables 1 and 2. Delays index is missing for observations in which any one of the 6 delays items is missing.

Supplement to: Rokicki S, Mwesigwa B, Waiswa P, Cohen J. Impact of solar light and electricity on the quality and timeliness of maternity care: a stepped-wedge cluster-randomized trial in Uganda. *Glob Health Sci Pract*. 2021;9(4). https://doi.org/10.9745/GHSP-D-21-00205

Supplement Table 12. Results of robustness checks

	All observed deliveries		Observed deliveries with some nighttime hours	
	**20-item index**	**36-item index**	**20-item index**	**36-item index**
**Panel A: Including only complete observations**				
Treated	2.955	4.015	3.987	4.574
	(-0.483, 6.392)	(1.016, 7.013)	(0.425, 7.548)	(1.445, 7.702)
Constant	42.916	54.258	42.418	53.930
	(41.487, 44.344)	(53.011, 55.505)	(41.079, 43.757)	(52.751, 55.109)
Observations	964	962	691	689
**Panel B: Multiple imputation of missing data[a]**				
Treated	3.162	4.161	4.191	4.778
	(-0.069, 6.392)	(1.336, 6.985)	(0.724, 7.657)	(1.737, 7.820)
Constant	42.610	54.145	42.162	53.817
	(41.327, 43.893)	(53.024, 55.267)	(40.897, 43.426)	(52.709, 54.926)
Observations	1118	1118	--[b]	--[b]
**Panel C: Dropping first 5 observations of an enumerator**				
Treated	3.933	4.743	5.013	5.043
	(0.920, 6.946)	(2.101, 7.386)	(1.627, 8.398)	(2.111, 7.975)
Constant	41.753	53.441	41.223	53.309
	(40.465, 43.040)	(52.309, 54.573)	(39.869, 42.578)	(52.132, 54.486)
Observations	990	990	648	648
**Panel D: Dropping first 5 observations of a provider[c]**				
Treated	3.836	5.205	4.600	5.298
	(0.691, 6.982)	(2.367, 8.043)	(0.959, 8.240)	(1.835, 8.762)
Constant	42.053	53.246	41.833	53.316
	(40.491, 43.616)	(51.837, 54.655)	(40.079, 43.588)	(51.655, 54.976)
Observations	595	595	366	366
**Panel E: Including multiple births and stillbirths (additional 15 observations)**				

Supplement to: Rokicki S, Mwesigwa B, Waiswa P, Cohen J. Impact of solar light and electricity on the quality and timeliness of maternity care: a stepped-wedge cluster-randomized trial in Uganda. *Glob Health Sci Pract*. 2021;9(4). https://doi.org/10.9745/GHSP-D-21-00205

Treated	3.083	4.190	4.019	4.618
	(-0.103,6.269)	(1.408,6.972)	(0.407,7.631)	(1.473,7.763)
Constant	42.594***	53.947***	42.433***	53.937***
	(41.345,43.843)	(52.856,55.037)	(41.133,43.733)	(52.804,55.069)
Observations	1133	1133	754	754

Notes: “Constant” indicates the mean outcome in facilities without the intervention. “Treated” indicates the change in average outcome due to the intervention. [a]Imputation conducted using iterative Markov chain Monte Carlo method assuming an underlying multivariate normal model and performing 10 imputed datasets. [b]Number of observations varies across imputations, between 792-801. Regressions additionally control for number of paid days spent at facility. [c]The observation is assigned to the provider who provided the most care from arrival to 1 hour postpartum (79% of observations have only one provider present throughout the observation).

Supplement to: Rokicki S, Mwesigwa B, Waiswa P, Cohen J. Impact of solar light and electricity on the quality and timeliness of maternity care: a stepped-wedge cluster-randomized trial in Uganda. *Glob Health Sci Pract*. 2021;9(4). https://doi.org/10.9745/GHSP-D-21-00205

Supplement Table 13. Patient and health worker characteristics before and after intervention deployment installation [mean (SD)]

	Before Intervention n=30	After Intervention n=30	p-value
**Provider-level characteristics**			
Average provider age (years)	34 (5)	33 (4)	0.34
Average provider years of experience	8 (6)	7 (6)	0.46
Average % of providers with Secondary education	0.02 (0.08)	0.01 (0.03)	0.28
Average % of providers with Certificate	0.59 (0.33)	0.63 (0.30)	0.68
Average % of providers with Diploma	0.39 (0.33)	0.37 (0.30)	0.83
Average % of providers in Officer position	0.13 (0.18)	0.12 (0.20)	0.92
Average % of providers in Midwife position	0.72 (0.26)	0.70 (0.26)	0.72
Average % of providers in Assistant position	0.15 (0.21)	0.18 (0.25)	0.62
**Patient-level characteristics**			
Average mother's age (years)	24.5 (2.2)	24.0 (1.9)	0.32
Average mother's parity	2.3 (0.6)	2.2 (0.6)	0.58
Average gestational age (weeks)	38.0 (0.8)	38.1 (0.7)	0.75

Notes: P-value from ANOVA test.

Supplement to: Rokicki S, Mwesigwa B, Waiswa P, Cohen J. Impact of solar light and electricity on the quality and timeliness of maternity care: a stepped-wedge cluster-randomized trial in Uganda. *Glob Health Sci Pract*. 2021;9(4). https://doi.org/10.9745/GHSP-D-21-00205

Supplement Table 14. Facilities with Solar Suitcase components in use during post-trial visits (n=30)

	Period 3 survey<br>Feb-April 2019	1st visit post RCT<br>June 2019	2nd visit post RCT<br>Oct 2019	3rd visit post RCT<br>Feb 2020<br>(1 year post trial)
All components	26 (87%)	23 (77%)	23 (77%)	18 (60%)
LED lights	30 (100%)	29 (97%)	29 (97%)	28 (93%)
Fetal doppler	29 (97%)	29 (97%)	28 (93%)	27 (90%)
Headlamps	29 (97%)	28 (93%)	27 (90%)	25 (83%)
USB port	28 (93%)	27 (93%)	28 (93%)	25 (83%)

Notes: "In-use" indicates that the component is functioning and available in the facility for use.
